# Outcomes and limitations of endoscopic ultrasound-guided hepaticogastrostomy in malignant biliary obstruction

**DOI:** 10.1186/s12876-021-01798-2

**Published:** 2021-05-05

**Authors:** Mateusz Jagielski, Michał Zieliński, Jacek Piątkowski, Marek Jackowski

**Affiliations:** Department of General, Gastroenterological and Oncological Surgery, Collegium Medicum Nicolaus Copernicus University, 53-59 Św. Józefa St, 87-100 Toruń, Poland

**Keywords:** Biliary obstruction, Hepaticogastrostomy, Endoscopic ultrasound, Interventional endoscopy

## Abstract

**Background:**

Transpapillary biliary drainage in ERCP is an established method for symptomatic treatment of patients with unresectable malignant biliary obstruction. Percutaneous transhepatic biliary drainage frequently remains the treatment of choice when the transpapillary approach proves ineffective. Recently, EUS-guided extra-anatomical anastomoses of bile ducts to the gastrointestinal tract have been reported as an alternative to percutaneous biliary drainage. To assess the usefulness of extra-anatomical intrahepatic biliary duct anastomoses to the gastrointestinal tract as endotherapy for unresectable malignant biliary obstruction and to determine factors affecting the efficacy of treatment.

**Methods:**

A prospective analysis of the treatment results of all patients with unresectable biliary obstruction treated with EUS-guided hepaticogastrostomy at our institution in the years 2016–2019.

**Results:**

Transmural intrahepatic biliary drainage (EUS-guided hepaticogastrostomy) was performed due to the ineffectiveness of ERCP in 53 patients (38 males, 15 females; mean age 74.66 [56–89] years) with unresectable biliary obstruction. Technical success of EUS-guided hepaticogastrostomy was achieved in 52/53 (98.11%) patients. Complications of endoscopic treatment were observed in 10/53 (18.87%) patients. Clinical success of EUS-guided hepaticogastrostomy was achieved in 46/53 (86.79%) patients. Bismuth type II–IV cholangiocarcinoma, hepatic metastases, ascites, suppurative cholangitis, and high blood bilirubin levels exceeding 30 mg/dL were independent factors for increased complications and inefficacy of EUS-guided hepaticogastrostomy.

**Conclusions:**

In the event of transpapillary biliary drainage proving ineffective, extra-anatomical anastomoses of intrahepatic bile ducts to the gastrointestinal tract provide an effective method for the treatment of patients with malignant biliary obstruction.

## Background

Endoscopic retrograde cholangiopancreatography (ERCP) with implantation of endoprosthesis for transpapillary biliary drainage is an established and widely used method for symptomatic treatment of patients with malignant biliary obstruction [1–3]. The efficacy rate for endoscopic bile duct stenting in this group of patients is high, with low and acceptable complication rates [3, 4]. Percutaneous transhepatic biliary drainage (PTBD) remains the treatment of choice when the transpapillary approach proves ineffective. However, PTBD is less effective and associated with higher complication rates than the transpapillary approach [5].

Recent decades have witnessed continuous advances in endoscopic ultrasound (EUS) [6], which facilitates direct, real-time visualization of structures surrounding the gastrointestinal tract [7]. As therapeutic uses of EUS continue to be developed, EUS-guided extra-anatomical bile duct anastomoses to the gastrointestinal tract has been reported as an alternative to PTBD in cases of ERCP failure [8–10]. Starting from initial publications describing EUS-guided transmural access to bile ducts, we have been witnessing continuous development of a method that facilitates a number of drainage techniques [11, 12]. Following the transmural bile duct puncture and establishment of transpapillary duodenal access, the procedure can be completed using a rendezvous technique. Alternatively, the stent can be deployed transpapillary using an antegrade technique [8–12]. In the absence of transpapillary access to the duodenum, a transmural bile ducts puncture, once performed, can be widened to form an anastomosis between the bile duct lumina and the gastrointestinal tract, and transmural prosthesis can be deployed to provide extra-anatomical biliary drainage [8–12]. EUS-guided transmural biliary drainage facilitates intrahepatic bile duct access via the esophagus or stomach (EUS-guided hepaticoesophagostomy or hepaticogastrostomy) or extrahepatic bile duct access via the duodenum (EUS-guided choledochoduodenostomy or cholecystoduodenostomy) [8–12].

As suggested by most publications about endoscopic biliary drainage, the choice of drainage technique and bile duct access should depend on anatomical conditions, tumor staging, and the experience of the treatment center [8–19]. No unified standards for the therapeutic management of patients subjected to EUS-guided endoscopic transmural biliary drainage are available in the current literature.

The objective of this study was to assess the usefulness of extra-anatomical anastomoses of intrahepatic biliary ducts to the gastrointestinal tract (EUS-guided hepaticogastrostomy) in the endoscopic treatment of unresectable malignant biliary obstruction and to determine factors affecting the efficacy of treatment.

## Methods

A prospective analysis of the treatment results of all consecutive obstructive jaundice patients with unresectable biliary obstruction, treated via transmural intrahepatic biliary drainage (EUS-guided hepaticogastrostomy), at a single institution, during the years 2016–2019, was performed.

The study was approved by the Ethics Committee of Institutional Review Board (Collegium Medicum Nicolaus Copernicus University) and proceeded in line with the tenets set by the Declaration of Helsinki. All patients gave their informed consent for endoscopic procedures.

Our clinic is a referral center that admits patients referred from other health centers. All patients with malignant biliary obstruction were assessed in detail by an interdisciplinary oncological team to determine further management.

### Inclusion criteria

Patients with obstructive jaundice caused by unresectable malignant biliary obstruction were qualified for the study based on clinical presentation (clinical symptoms, blood analyses, and imaging studies) and histopathological findings. Included in the study were adults (≥ 18 years) of both genders who had provided written consent to the proposed interventional treatment and in whom ERCP had either failed (i.e.,bile duct could not be catheterized despite three attempts at ERCP) or was deemed impossible due to the lack of access to the major duodenal papilla (i.e.,malignant peripapillary infiltration preventing localization of major duodenal papilla or duodenal obstruction due to advanced cancer).

### Exclusion criteria

Patients with obstructive jaundice caused by unresectable malignant biliary obstruction and with a surgical history involving the biliopancreatic area, a history of transmural/transduodenal extrahepatic biliary drainage (EUS-guided endoscopic choledochoduodenostomy or cholecystoduodenostomy) or percutaneous transhepatic biliary drainage were excluded from the study. Patients in whom EUS-guided hepaticogastrostomy was performed without the diagnosis of cancer, were also excluded from the study.

### Study group

The final study group consisted of patients in whom EUS-guided hepaticogastrostomy was performed because of unresectable malignant biliary obstruction and a lack of transpapillary access to the bile ducts in the course of ERCP.

### Algorithm for the type of EUS-guided transmural biliary drainage

In all patients before EUS-guided hepaticogastrostomy, in the course of endoscopic ultrasound examination technical conditions for EUS-guided choledochoduodenostomy or cholecystoduodenostomy were evaluated. In case of high failure risk or lack of required technical conditions for EUS-guided choledochoduodenostomy or cholecystoduodenostomy a decision was being taken of EUS-guided hepaticogastrostomy execution.

### Endoscopic procedures

**Fig. 1:**
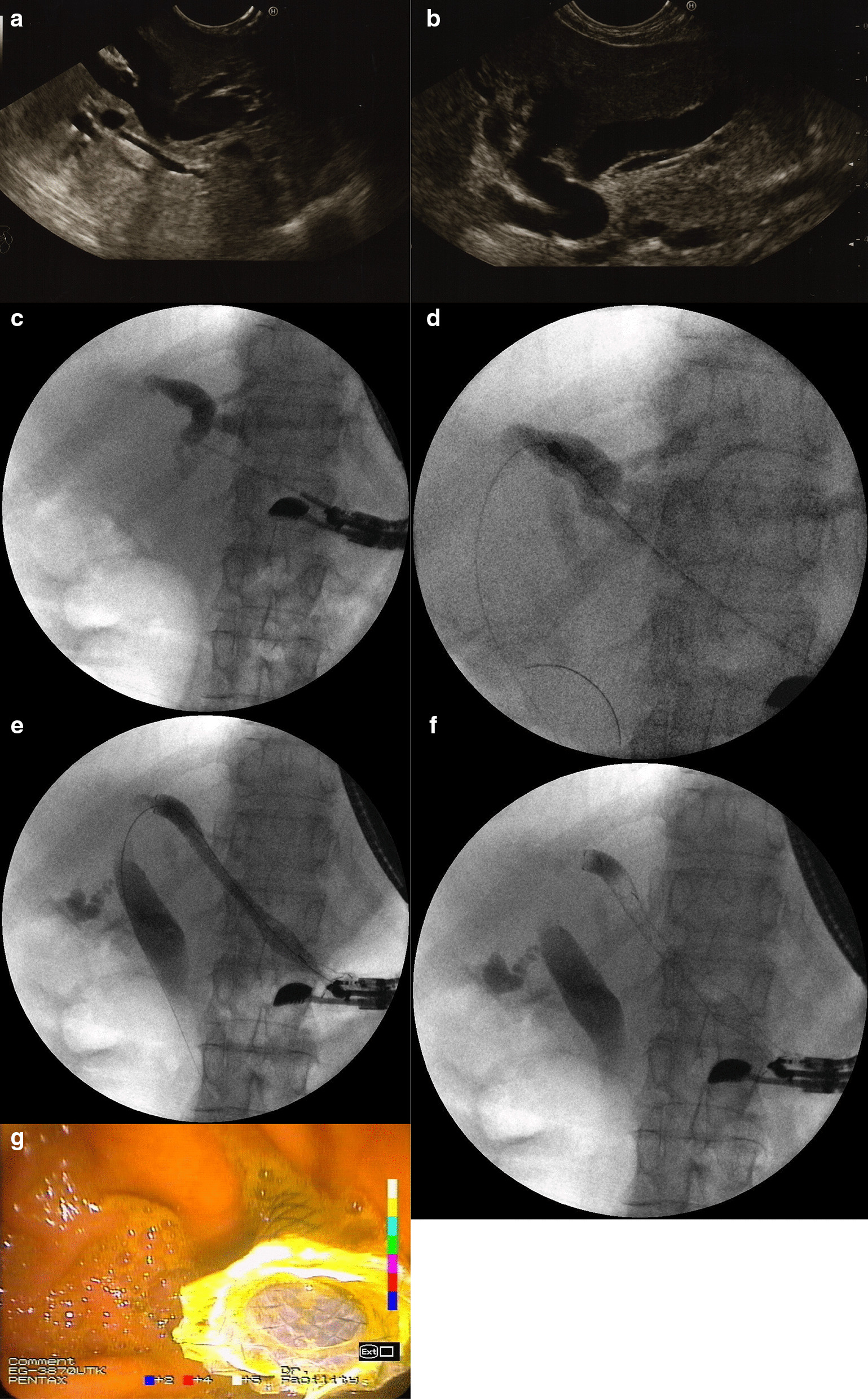
**a**–**g** EUS-guided hepaticogastrostomy in a patient with unresectable pancreatic head tumor (adenocarcinoma). Dilated bile ducts of left liver lobe visible in endosonographic image preceding endoscopic hepaticogastrostomy (**a**, **b**). Transmural puncture of the enlarged bile ducts within the left liver lobe was performed using a 19G needle and a contrast agent filled the enlarged bile ducts (**c**). A guidewire was introduced into the left bile duct and was directed towards the main bile duct. A 10 Fr cystostome was used to established a hepaticogastric fistula (**d**). Half-coated self-expandable endoprosthesis was introduced via the fistula (**e**–**g**)

EUS-guided hepaticogastrostomy procedures (Fig. 1a–g) were performed using a therapeutic linear array echoendoscope (EG38UT Pentax, Tokyo, Japan) under general anesthesia. Prophylactic antibiotic therapy (Ciprofloxacin 400 mg IV) was administered to all patients prior to the endoscopic procedure.

During EUS-guided transmural/transgastric hepaticogastrostomy, a linear array echoendoscope was introduced into the stomach. The intrahepatic biliary ducts within the left lobe segments II and III were usually revealed on endosonographic imaging within the subcardial region on the lesser curvature side. Color Doppler ultrasound was used prior to performing an EUS-guided puncture through the stomach wall to confirm the absence of vascular structures in the potential puncture line. The enlarged biliary ducts within the left liver lobe (up to a diameter of ≥ 5 mm) were punctured using a 19G needle (EchoTip Ultra 19, Cook Medical, Bloomington, Indiana, USA) under endosonographic control. Following stylet removal, bile content aspiration was performed to confirm an intraductal needle tip location. The aspired bile was sent for bacteriological assays. Next, the contrast agent was administered via the intraductal needle under fluoroscopic control to obtain an antegrade cholangiogram. After flushing the needle with physiological saline, a rigid 0.035-inch guidewire (Dreamwire;Boston Scientific Corp.,Marlborough, Massachusetts, USA) was introduced through the needle lumen into the bile duct. The guidewire was introduced into the left bile duct and then directed towards the common bile duct with the intention of gaining access to the duodenal lumen so as to continue the procedure using the rendezvous approach, or to perform antegrade deployment of the transpapillary stent. Following several unsuccessful attempts to access the duodenum due to malignant stricture of the main bile duct or duodenum, the needle was withdrawn, while the position of the guidewire was maintained and a hepaticogastric fistula was established using a 10 Fr cystostome (Cook Medical, USA). Half-coated (i.e. non-coated in the intrahepatic segment) self-expanding endoprosthesis (Giobor, diameter of 10 mm, length of 8 or 10 cm; Taewoong Medical,Gyeonggi-do,Korea) was introduced through the newly formed fistula under endosonographic and fluoroscopic guidance. The catheter was then introduced through the endoprosthesis into the bile duct, and a contrast agent was administered for a follow-up cholangiographic examination to confirm correct positioning of the transmural endoprosthesis, correct biliary drainage, and the absence of any leaks from the biliary tract.

### Conservative treatment and monitoring

In the group of patients with suppurative cholangitis, empirical intravenous antibiotic therapy was initially continued in the hospital setting post-operatively then switched to targeted antibiotic therapy after the susceptibility test results were obtained for bacteria cultured from bile aspired during the endoscopic procedure. If no further hospitalization was required, patients who had undergone EUS-guided hepaticogastrostomy were discharged after a downward trend was observed for cholestasis parameters in blood tests (usually on day two after the endoscopic procedure). After discharge from the clinic, regular blood tests were performed to assess cholestasis parameters. Initially, these were performed weekly for the first month after the treatment. After this period, follow-up examinations and outpatient visits within the Surgery Clinic or the Oncology Clinic were scheduled on a case-by-case basis.

### Definitions

Technical success was defined as successful (as determined by endoscopic and radiological imaging) placement of a transmural stent, with the distal end located within the lumen of the biliary duct and the proximal end being located within the lumen of the gastrointestinal tract (stomach). The technical success of the procedure was confirmed by unobstructed flow of the contrast agent along the transmural stent from the bile duct into the stomach with no leaks outside the biliary tract or the transmural stent.

Clinical success was defined as the absence of clinical features of mechanical obstruction within the bile ducts and a decrease in the parameters of cholestasis in laboratory blood tests. An 80% reduction in the bilirubin level compared to the baseline, as determined two weeks after the endoscopic procedure, was required to confirm the clinical success.

Complications of endoscopic treatment were divided into early complications (occurring up to 30 days after treatment), evaluated in line with the Clavien-Dindo classification [13], and late complications (occurring more than 30 days after treatment).

Periprocedural mortality was defined as death within 30 days after endoscopic treatment.

### Statistical analysis

All statistical calculations were performed using StatSoft statistical package Inc. data analysis software system version 12.0 (2014, STATISTICA, Tulsa, Oklahoma, USA). Quantitative variables were characterized by arithmetic means, minimal and maximal values (range). Qualitative data were presented as means and percentages. To verify if quantitative variable came from a normally distributed population, the Shapiro–Wilk test was used. To for equality of variance, the Levene’s (Brown–Forsythe) test was used. Significances in differences between two groups (independent variables model) were analyzed using Student’s t-test (Welch’s t-test in case of unequal variances) or Mann–Whitney U test (when Student’s t-test was not applicable or for variables measured with ordinal scale). Significances in differences between more than two groups were checked with the F (ANOVA) or Kruskal–Wallis test (in case of failure to meet the applicability conditions of ANOVA). When statistically significant differences were obtained between groups, post hoc tests were used (Tukey's test for F, Dunn's test for Kruskal–Wallis test). In cases of models of two related variables, the Student's t-test or the Wilcoxon-pair-order test (in case of failure to meet the applicability conditions of the Student's t-test or for variables measured on an ordinal scale) was used. The significance of differences between more than two in the model of related variables was checked by analysis of variance with repeated measures or Friedman's test (in case of failure to meet the applicability conditions of ANOVA with repeated measures or for variables measured on an ordinal scale). The chi-squared test of independence was used for qualitative variables (with Yates’s correction for continuity when the cell number was less than 10, with Cochran’s condition checked and Fisher’s exact test). In all calculations, significance was assumed if P < 0.05.

## Results

A total of 584 patients with obstructive jaundice caused by unresectable malignant biliary obstruction underwent endoscopic treatment at our institution within the years 2016–2019.

In 526/584 (90.07%) patients, effective biliary tract stenting across the major duodenal papilla was achieved via ERCP. The remaining 58/584 (9.93%) patients were recommended alternative bile duct drainage as a result of ERCP being inefficient or being deemed impossible to perform due to neoplastic or surgical remodeling of anatomy. EUS-guided hepaticogastrostomy was performed in 53/58 (91.39%) patients, EUS-guided choledochoduodenostomy was performed in 2/58 (3.45%) patients, EUS-guided cholecystoduodenostomy was performed in 1/58 (1.72%) patient, percutaneous transhepatic biliary drainage was performed in 1/58 (1.72%) patient, and endoscopic hepaticojejunostomy was performed in 1/58 (1.72%) patient after complete stomach resection.

In relation to all ERCP procedures performed in years 2016–2019, ERCP procedure failures were observed in 58/2461 (2.36%) patients.

### Patient characteristics

In the 53 patients (38 men, 15 women; mean age 74.66 [56–89] years) with unresectable malignant biliary obstruction, EUS-guided hepaticogastrostomy was performed due to ERCP inefficacy (bile duct catheterization failing despite three attempts at ERCP) in 5/53 (9.43%) patients, due to ERCP being deemed impossible (malignant infiltration of duodenal wall in the peripapillary region preventing localization of the major duodenal papilla in 23/53 [43.39%] patients, and due to duodenal obstruction in the course of cancer in 25/53 [47.18%] patients). In all cases with duodenal obstruction its level was located in the area of superior flexure of duodenum, which prevented insertion of duodenoscope into descending part of duodenum.

Detailed clinical characteristics of the patients are presented in Tables 1 and 2.

**Table 1:** Detailed The clinical characteristics of all 53 patients underwent EUS-guided hepaticogastrostomy

Male gender, n (%)	38 (71.70%)
Age, mean [range]	74.66 [56–89]
Biliary obstruction cause, n (%)	
Pancreatic cancer	19 (35.8%)
Cholangiocarcinoma	14 (26.4%)
Gallbladder cancer	6 (11.3%)
Hepatocellular carcinoma	3 (5.7%)
Major duodenal papillary cancer	6 (11.3%)
Duodenal cancer	1 (1.9%)
Metastatic colorectal cancer	2 (3.8%)
Metastatic breast cancer	1 (1.9%)
Metastatic cancer of unknown origin	1 (1.9%)
Ascites, n (%)	11 (20.75%)
Liver metastases, n (%)	14 (26.42%)
Suppurative cholangitis, n (%)	21 (39.62%)
Reason for EUS-guided hepaticogastrostomy., n (%)	
Duodenal obstruction	25 (47.18%)
Periampullary tumor infiltration	23 (43.39%)
Failed biliary cannulation	5 (9.43%)

**Table 2:** Laboratory data of patients underwent EUS-guided hepaticogastrostomy

Parameter in blood test	Result
Hemoglobin, g/dl, mean, (SD) [range]	13.2 (2.1) [9.2–19.4]
Leukocytes, mm^3^, mean, (SD) [range]	13.2 (7.4) [4.5–36.7]
Thrombocytes, mm^3^, mean, (SD) [range]	292.9 (125.9) [110.0–555.0]
C-reactive protein, mg/L, mean, (SD) [range]	119.0 (133.3) [11.4–555.1]
Procalcitonin, µg/L, mean, (SD) [range]	8.9 (22.5) [0.1–111.6]
Creatinine, mg/dl, mean, (SD) [range]	1.5 (0.8) [0.4–4.4]
Bilirubin, mg/dl, mean, (SD) [range]	21.0 (9.5) [5.9–42.5]
AST, U/L, mean, (SD) [range]	405.5 (265.3) [81.0–1109.0]
ALT, U/L, mean, (SD) [range]	406.0 (251.2) [90.0–1029.0]
GGT, U/L, mean, (SD) [range]	1 858.2 (520.0) [1045.0–3098.0]
ALP, U/L, mean, (SD) [range]	1 868.8 (586.0) [146.0–3340.0]
INR, mean, (SD) [range]	1.3 (0.3) [0.9–1.8]

*AST* aspartate aminotransferase, *ALT* alanine aspartate aminotransferase, *GGT* gamma-glutamyltransferase, *ALP* alkaline phosphatase, *INR* international normalized ratio

In 4/53 (7.56%) patients with unresectable malignant biliary obstruction, indication for EUS-guided hepaticogastrostomy were liver metastases lesions, which were cause of compression on bile ducts (especially in left liver lobe) and led to prestenotic dilation of bile ducts with symptoms of mechanical jaundice. Therefore, the liver metastases lesions were indication for endoscopic palliative biliary drainage. In the remaining 14/53 (26.42%) patients with liver metastasis, metastatic cancer was no indication to begin endoscopic treatment, because metastasis did not compress the bile ducts and did not lead to prestenotic dilation of bile ducts.

### Technical data of endoteraphy

Technical success of extra-anatomical endoscopic anastomosis of intrahepatic bile ducts to the stomach was achieved in 52/53 (98.11%) patients. The mean duration of the endoscopic procedure was 34 (11–84) minutes. The average number of transmural punctures during the procedure was 1.36 (1–4). The mean size of the punctured intrahepatic duct was 12.79 mm (5–21 mm). The mean distance between the stomach lumen and the punctured duct lumen was 22.74 [10–33] mm. Bile ducts punctured for anastomosis were located within liver segments III and II in 46 and 7 patients, respectively. The mean duration of hospital stay was 3.44 (2–8) days.

### Early complications

Complications of endoscopic treatment were observed in 10/53 (18.87%) patients. Early complications of endotherapy were observed in 7/53 (13.21%) patients. Bleeding into the upper part of the gastrointestinal tract, requiring conservative treatment using packed red blood cells and fresh frozen plasma transfer (Clavien–Dindo grade II), was observed in two patients. Postoperative biliary sepsis, requiring intravenous broad-spectrum antibiotic therapy (Clavien–Dindo grade II), was observed in one patient.

### Mortality

The periprocedural mortality (Clavien-Dindo grade V) rate was 4/53 (7.55%). In three patients, death was due to biliary peritonitis caused by bile leakage from the hepaticogastric anastomosis. In one patient, death was due to biliary sepsis.

### Late complications

Late endoscopic treatment complications manifested as transmural stent obstruction in 3/53 (5.66%) patients. During the course of long-term follow-up, 3/53 (5.66%) patients required repeated endoscopic procedures due to transmural stent obstruction caused by hyperplastic cancer tissue. No evidence of transmural stent migration was observed within the long-term follow-up period for any patient.

### Outcomes of endoteraphy

Clinical success of EUS-guided hepaticogastrostomy was achieved in 46/53 (86.79%) patients. In 35/53 (66.04%) patients, chemotherapy could be administered following endoscopic procedure due to blood bilirubin levels dropping below the threshold that facilitates chemotherapy. The mean duration of follow-up was 155 (8–434) days.

Detailed technical data concerning the endoscopic procedure and clinical outcomes of endoteraphy are presented in Table 3.

**Table 3:** Detailed technical data and clinical outcomes of EUS-guided hepaticogastrostomy

Factors	All (n = 53)
Procedure time, min, mean, (SD), [range]	31.2 (15.0) [11–84]
Diameter of the punctured intrahepatic duct, mm, mean, (SD), [range]	12.79 (4.8) [5–21]
Distance between the stomach and the punctured duct, mm, mean, (SD), [range]	22.74 (8.0) [10–33]
Side of puncture, n, (%)	
Liver segment II	7 (13.21%)
Liver segment III	46 (86.79%)
Number of puncture, n, (%)	
1	32 (60.38%)
2	13 (24.53%)
3	3 (5.66%)
4	5 (9.43%)
Technical success, n, (%)	52/53 (98.11%)
Complications of endoscopic treatment, n, (%)	10/53 (18.87%)
Early complications	7 (13.21%)
Late endoscopic treatment complications	3 (5.66%)
The periprocedural mortality	4 (7.55%)
Clinical success of EUS-guided hepaticogastrostomy, n, (%)	46/53 (86.79%)

### Negative predictors for the efficacy of EUS-guided hepaticogastrostomy

Logistic regression analysis was used to identify independent risk factors for complications and inefficacy of EUS-guided hepaticogastrostomy. These included: Bismuth type II–IV cholangiocarcinoma (P = 0.0023, HR = 0.05, 95% CI 0.01–0.35), hepatic metastases (P = 0.0093, HR = 0.05, 95% CI 0.01–0.48), ascites (P = 0.0157, HR = 0.11, 95% CI 0.02–0.66), suppurative cholangitis (P = 0.0016, HR = 0.03, 95% CI 0.01–0.25), and high blood bilirubin levels exceeding 30 mg/dL (P = 0.0010, HR = 0.02, 95% CI 0.01–0.21). Other independent risk factors included: the size of the punctured bile duct being less than 7 mm (P = 0.0190, HR = 2.12, 95% CI 1.13–3.98), the duration of the procedure being longer than 40 min (P = 0.0013, HR = 0.87, 95% CI 0.79–0.95), and more than two biliary punctures performed during the endoscopic procedure prior to the establishment of hepaticogastric anastomosis (P = 0.0007, HR = 0.25, 95% CI 0.11–0.56). The distance between the stomach lumen and the drained bile duct lumen was not shown to affect the efficacy of endotherapy (P = 0.6773, HR = 1.02, 95% CI 0.93–1.13).

## Discussion

Most publications available either deal with the outcomes of endoscopic drainage of extrahepatic bile ducts being achieved by means of choledochoduodenostomy/cholecystoduodenostomy or presenting combined outcomes of transmural biliary drainage from extra- and intrahepatic access [11, 12, 14–16]. This makes it difficult to compare the results of this study with those obtained by others. This prospective study showed that transgastric drainage of intrahepatic bile ducts (EUS-guided hepaticogastrostomy) in patients with malignant biliary obstruction following ERCP failure is an effective endotherapeutic modality with an acceptable complication rate, and may be an alternative method for minimally invasive treatment for these patients. Notably, all patients in the study had cancer within the biliopancreatic area, which increased complication risk as well as periprocedural mortality. The prognosis was further worsened by cancer comorbidities, mainly cancer-related cachexia. However, the good results of endoscopic treatment support the efficacy of extra-anatomical transmural biliary tract anastomoses.

In most institutions, PTBD remains the treatment of choice when a transpapillary approach proves ineffective [5, 20]. However, PTBD is less effective and is associated with higher complication rates than the transpapillary approach [5]. In addition, external percutaneous drainage remains a persistent problem in long-term palliative care as it often adds to the patient’s discomfort [5]. Compared to conventional percutaneous biliary drainage, endoscopic transmural anastomoses between the biliary and gastrointestinal tracts are characterized by similar technical and clinical success rates of more than 90%, but with complication rates being significantly higher in the external drainage group [20, 21]. In their systematic review and meta-analysis of nine studies, Sharaiha et al. demonstrated no difference in technical success rates between endoscopic extra-anatomical bile duct anastomoses and external percutaneous drainage in patients following ERCP [22]. The same study revealed a better clinical success rate as well as a lower number of complications and reinterventions for transmural endoscopic anastomoses compared to percutaneous drainage [22]. In addition to the reduction of the above-mentioned discomfort in palliative care, the superiority of endoscopic bile duct anastomoses over percutaneous drainage consists mainly of its reduction in post-procedural risk for infections, which frequently require reinterventions and hospitalizations in patients with percutaneous drainage [22].

Four meta-analyses available in the literature on the subject of EUS-guided extra-anatomical bile duct anastomoses revealed high technical (90%–94.7%) and clinical success (87%–94%) rates, with an acceptable complication rate of 16%–29% [11, 14–16]. When comparing extrahepatic biliary tract access, via choledochoduodenostomy/cholecystoduodenostomy, to intrahepatic access, via hepaticogastrostomy, the technical and clinical success rates are similar. Whereas a higher number of complications are observed in patients with intrahepatic access [11, 17]. On the other hand, a systematic review and meta-analysis carried out by Uemura et al. did not reveal any differences in the efficacy and safety of EUS-guided hepaticogastrostomy compared to EUS-guided choledochoduodenostomy/cholecystoduodenostomy [18].

In experienced interventional endoscopic centers, when making a choice regarding the type and technique for extra-anatomical transmural biliary drainage, one should take into consideration the treatment center experience and the estimated complication risks that are frequently related to anatomical conditions and cancer stage [19]. Intrahepatic access to the biliary tract via hepaticogastrostomy is generally considered to be technically more challenging than extrahepatic access via choledochoduodenostomy/cholecystoduodenostomy. Consequently, EUS-guided hepaticogastrostomy is reserved for patients in whom choledochoduodenostomy/ cholecystoduodenostomy is considered impossible [19]. On the other hand, of all the techniques for extra-anatomical transmural endoscopic biliary drainage, hepaticogastrostomy has the broadest range of clinical indications [14–16]. Neither duodenal obstruction, biliary obstruction at the hilar level, nor alterations of gastrointestinal anatomy following previous surgical procedures preventing transduodenal drainage of extrahepatic bile ducts, are contraindications for EUS-guided hepaticogastrostomy [14–16].

EUS-guided hepaticogastrostomy is an extra-anatomical transmural endoscopic biliary drainage modality that is most frequently performed at our center, not only because of our experience, but also because of its high efficacy combined with a relatively low complication rate in our experienced center. In our opinion, EUS-guided hepaticogastrostomy is not only an alternative to be used following failed attempts at ERCP, but may also be used as first-line treatment in the endotherapy of unresectable malignant biliary obstruction in experienced interventional endoscopic centers.

In experienced institutions, EUS-guided hepaticogastrostomy in patients with obstructive jaundice secondary to malignant biliary obstruction has an efficacy rate similar to that of ERCP [23]. Three randomized studies compared the results of patients with malignant biliary obstruction involving transpapillary drainage treated with ERCP vs EUS-guided transmural biliary drainage [24–26]. No differences in the efficacy or safety of both treatments were observed in two studies [24, 25]. In contrast, the study by Paik et al. also failed to reveal any differences in the efficacy of treatment, but demonstrated that extra-anatomical transmural anastomoses were associated with lower complication rates compared to ERCP [26]. In theory, EUS-guided extra-anatomical transmural anastomoses between the biliary and gastrointestinal tracts, compared to transpapillary drainage via ERCP, may prevent injuries to the major duodenal papilla, thus reducing acute pancreatitis risk [27, 28]. There is also less contact between the endoprosthesis and tumor tissues, reducing the risk of the transmural stent becoming overgrown and obstructed by cancer tissue. Thus, the transmural self-expandable stents used in EUS-guided hepaticogastrostomy should remain patent longer than self-expandable stents introduced via the transpapillary route in the course of ERCP procedures. This is particularly important in cases of distal malignant bile duct stenosis, where transmural prostheses are usually not in direct contact with neoplastic tissue. On the other hand, this is not valid for of Bismuth type II–IV hilar tumors, where the transmural stents installed to drain the right liver lobe splint the malignant stricture. In our study, it was in patients with bile duct malignancies involving the liver hilum where increased rates of repeated endoscopic interventions were observed as the result of self-expandable transmural stent obstructions. In reinterventions, stent patency was restored using another fully coated self-expandable stent introduced into the lumen of the occluded stent. In addition, obstruction of the transmural stents frequently led to suppurative cholangitis. As a result, nasobiliary drainage had to be temporarily installed within the transmural stent in some patients for active drainage of bile during the course of reintervention.

This study found negative predictors for the efficacy of EUS-guided hepaticogastrostomy including, in addition to the aforementioned technical conditions of the procedure itself. These were: Bismuth type II–IV cholangiocarcinoma, hepatic metastases, ascites, suppurative cholangitis, and high blood bilirubin levels exceeding 30 mg/dL. Bismuth type II–IV cholangiocarcinoma was a negative predictive factor for endoscopic procedure efficacy and was not related to the lack of adequate drainage in our patients. In all patients whose malignant lesion involved the liver hilum, access to the right intrahepatic duct was gained via the stricture being splinted by a stent introduced into the left intrahepatic duct via the stomach, as previously described [29, 30]. The presence of metastatic lesions in the liver and high blood bilirubin levels also had a negative effect on treatment outcomes. Both findings might have had a common denominator. The high blood bilirubin level may have been due to hepatic parenchyma being damaged secondary to the presence of metastatic lesions rather than by bile duct obstruction alone. Ascites was another negative predictor of endotherapeutic success. The presence of ascitic fluid between the gastric wall and the liver not only makes it technically difficult to perform a transgastric puncture of the enlarged bile ducts due to the increased distance between the bile ducts and the gastrointestinal tract, but also makes it difficult to maintain the transmural stent in a correct and stable position, increasing the risk of stent migration and consequently, bile leakage from the anastomosis into the peritoneum.

Based on these factors, it appears that the best treatment results can be obtained in patients with distal biliary stricture, no intrahepatic metastatic lesions, blood bilirubin levels < 30 mg/dL, and no signs of cholangitis or ascites.

Our study has some limitations which should be considered when interpreting our findings. The main limitations of this study include the lack of randomization and the fact that the study was performed only on a selected group of patients from a single center. Moreover, cost analysis was not performed and a long follow-up was not assessed, which are additional limitations of our study.

The current literature does not provide a unified standard for the therapeutic management regarding EUS-guided endoscopic transmural biliary drainage due to inefficacy or failure of transpapillary drainage attempted in the course of ERCP in patients with obstructive jaundice secondary to unresectable malignant biliary obstruction. Consequently, further studies on the management of these patients are recommended. As suggested by our results, in the event of transpapillary biliary drainage proving ineffective, extra-anatomical bile duct anastomoses to the gastrointestinal tract provides an effective method in patients with malignant biliary obstruction. Furthermore, in experienced sites, the efficacy of EUS-guided hepaticogastrostomy is similar to that of transpapillary drainage in the course of ERCP. Compared to the latter, EUS-guided hepaticogastrostomy has a wider range of indications in patients with obstructive jaundice secondary to unresectable malignant biliary obstruction and can be used as the first-line treatment in these patients. Nevertheless, further studies are now necessary in order to evaluate the efficacy of this treatment strategy in detail.

## Data Availability

The datasets used and analyzed during the current study available from the corresponding author on reasonable request.

## Abbreviations

ERCP: Endoscopic retrograde cholangiopancreatography
PTBD: Percutaneous transhepatic biliary drainage
EUS: Endoscopic ultrasound
